# Sequence Divergence and Retrotransposon Insertion Underlie Interspecific Epigenetic Differences in Primates

**DOI:** 10.1093/molbev/msac208

**Published:** 2022-10-11

**Authors:** Mayu Hirata, Tomoko Ichiyanagi, Hirokazu Katoh, Takuma Hashimoto, Hikaru Suzuki, Hirohisa Nitta, Masaki Kawase, Risako Nakai, Masanori Imamura, Kenji Ichiyanagi

**Affiliations:** Laboratory of Genome and Epigenome Dynamics, Department of Animal Sciences, Graduate School of Bioagricultural Sciences, Nagoya University, Nagoya 464-8601, Japan; Laboratory of Genome and Epigenome Dynamics, Department of Animal Sciences, Graduate School of Bioagricultural Sciences, Nagoya University, Nagoya 464-8601, Japan; Laboratory of Genome and Epigenome Dynamics, Department of Animal Sciences, Graduate School of Bioagricultural Sciences, Nagoya University, Nagoya 464-8601, Japan; Laboratory of Genome and Epigenome Dynamics, Department of Animal Sciences, Graduate School of Bioagricultural Sciences, Nagoya University, Nagoya 464-8601, Japan; Laboratory of Genome and Epigenome Dynamics, Department of Animal Sciences, Graduate School of Bioagricultural Sciences, Nagoya University, Nagoya 464-8601, Japan; Laboratory of Genome and Epigenome Dynamics, Department of Animal Sciences, Graduate School of Bioagricultural Sciences, Nagoya University, Nagoya 464-8601, Japan; Laboratory of Genome and Epigenome Dynamics, Department of Animal Sciences, Graduate School of Bioagricultural Sciences, Nagoya University, Nagoya 464-8601, Japan; Molecular Biology Section, Department of Cellular and Molecular Biology, Center for the Evolutionary Origins of Human Behavior, Kyoto University, Inuyama, Aichi 484-8506, Japan; Molecular Biology Section, Department of Cellular and Molecular Biology, Center for the Evolutionary Origins of Human Behavior, Kyoto University, Inuyama, Aichi 484-8506, Japan; Laboratory of Genome and Epigenome Dynamics, Department of Animal Sciences, Graduate School of Bioagricultural Sciences, Nagoya University, Nagoya 464-8601, Japan

**Keywords:** epigenome, evolution, primate, transcription factor, retrotransposon

## Abstract

Changes in the epigenome can affect the phenotype without the presence of changes in the genomic sequence. Given the high identity of the human and chimpanzee genome sequences, a substantial portion of their phenotypic divergence likely arises from epigenomic differences between the two species. In this study, the transcriptome and epigenome were determined for induced pluripotent stem cells (iPSCs) generated from human and chimpanzee individuals. The transcriptome and epigenomes for trimethylated histone H3 at lysine-4 (H3K4me3) and at lysine-27 (H3K27me3) showed high levels of similarity between the two species. However, there were some differences in histone modifications. Although such regions, in general, did not show significant enrichment of interspecies nucleotide variations, gains in binding motifs for pluripotency-related transcription factors, especially POU5F1 and SOX2, were frequently found in species-specific H3K4me3 regions. We also revealed that species-specific insertions of retrotransposons, including the LTR5_Hs subfamily in human and a newly identified LTR5_Pt subfamily in chimpanzee, created species-specific H3K4me3 regions associated with increased expression of nearby genes. Human iPSCs have more species-specific H3K27me3 regions, resulting in more abundant bivalent domains. Only a limited number of these species-specific H3K4me3 and H3K27me3 regions overlap with species-biased enhancers in cranial neural crest cells, suggesting that differences in the epigenetic state of developmental enhancers appear late in development. Therefore, iPSCs serve as a suitable starting material for studying evolutionary changes in epigenome dynamics during development.

## Introduction

Humans and chimpanzees share approximately 98–99% identity in their genomic sequences (Chimpanzee Sequencing Analysis Consortium 2005), but they show many phenotypic differences (Varki 2000; Varki and Altheide 2005; Somel et al. 2013). It has been shown that small changes in the amino acid sequence of proteins, as well as gains of new proteins in one species, created these interspecific differences; the former is exemplified by sequence changes in FOXP2 (Enard et al. 2002), while the latter by the emergence of NOTCH2NL in the human lineage (Fiddes et al. 2018; Suzuki et al. 2018). On the other hand, it is also considered that interspecific differences can arise from changes in gene expression patterns (King and Wilson 1975; Caceres et al. 2003; Carroll 2005), which could arise from genetic changes in *cis*-regulatory elements, such as enhancers. Gene expression is regulated by epigenetic modifications, such as methylation and acetylation of histone proteins and methylation of DNA, in regulatory regions and gene bodies. Deposition of some chromatin modifications, such as histone H3 acetylation at lysine-27 (H3K27ac) in a given nucleosome, is dictated by binding of transcription factors (TFs) and co-activators, at or near the regions, whereas deposition of some others, such as dimethylation of histone H3 at lysine-9 (H3K9me2), seems to be more independent of genetic sequence and more dependent on chromatin environments in the nuclear space. It is conceivable that, with or without changes in the underlying DNA sequence, interspecific differences in epigenetic modifications play an important role in the divergence of the transcriptome and phenotype. To examine this possibility and understand the underlying mechanisms, it is important to elucidate the conditions (or requirements) for epigenetic diversification between closely related species.

The DNA methylation profiles have been compared among human, chimpanzee, and other primates (Enard et al. 2004; Farcas et al. 2009; Pai et al. 2011; Zeng et al. 2012; Fukuda et al. 2013, 2017; Gallego Romero et al. 2015), which revealed that differential DNA methylation is an important molecular mechanism driving the divergence of gene expression levels and alternative splicing patterns involved in disease vulnerabilities. Some of these differences in DNA methylation arise from genetic changes, such as those in TF-binding sites (TFBSs) and insertion of retrotransposons (Fukuda et al. 2017). A previous report (Prescott et al. 2015) compared the patterns of H3K27ac and associated open chromatin states between human and chimpanzee cranial neural crest cells (CNCCs) that were derived from induced pluripotent stem cells (iPSCs), and revealed that many of the changes in enhancer activity are associated with changes in the underlying genetic sequence. It has recently been shown that structural variations (insertions, deletions, and inversions) in genomes contribute to interspecies differences in active chromatin marks, such as histone H3 trimethylation at lysine-4 (H3K4me3) (Zhuo et al. 2020). However, not all epigenetic changes can be explained in terms of genetic changes, leaving a possibility for changes in the epigenetic program during development. With respect to the repressive states of chromatin, transposable elements (TEs) are marked similarly with histone H3 trimethylation at lysine-9 (H3K9me3) in human and chimpanzee iPSCs (Ward et al. 2018). Despite the strong association between TEs and H3K9me3, TE transpositions do not induce the silencing of neighboring genes at the new insertion site (Ward et al. 2018). Histone H3 trimethylation at lysine-27 (H3K27me3) is a repressive mark associated with gene promoters, and it was reported that, in iPSCs, more H3K27me3 peaks are present in human than chimpanzee, whereas more H3K27ac peaks are present in chimpanzee than human (Gallego Romero et al. 2015), suggesting differences in gene regulation.

We have established chimpanzee iPSCs (two from females and one from a male) (Kitajima et al. 2020). These show a colony morphology similar to that of human iPSCs, the same pluripotent state (called a primed state), and an ability to form neurospheres in a manner similar to the neurosphere formation by human iPSCs, thus offering an opportunity to study the developmental dynamics of the epigenome and its differences between human and chimpanzee. In this study, messenger RNA sequencing (mRNA-seq) and chromatin immunoprecipitation sequencing (ChIP-seq) for active and repressive histone modifications, H3K4me3 and H3K27me3, respectively, were performed to determine the transcriptomes and chromatin states in chimpanzee and human iPSCs for interspecific comparison. While the transcriptome and epigenome profiles were highly conserved between the two species, there were differences in the histone modifications, some of which were associated with the transcriptional divergence. The origins of the epigenetic differences are discussed based on the differences in the underlying genetic sequence, including base substitutions and species-specific TE insertions.

## Results

### The Gene Expression Patterns Are Highly Similar between Human and Chimpanzee iPSCs

**Fig. 1.**
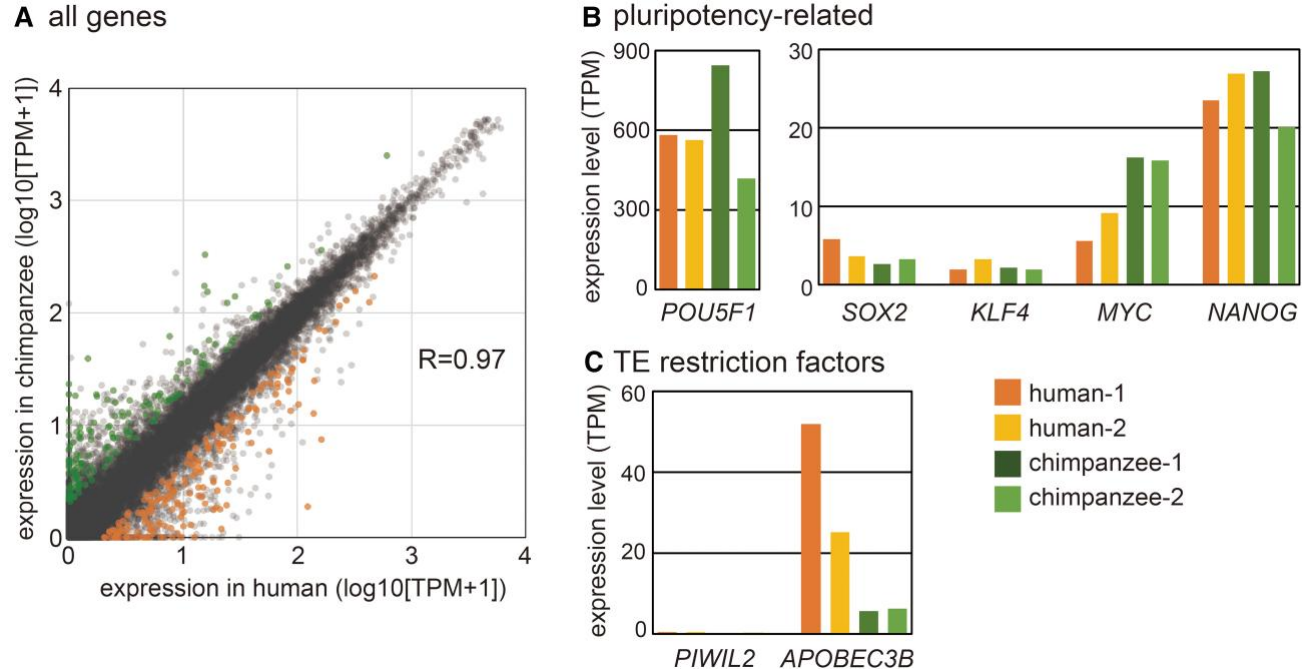
Comparison of gene expression between human and chimpanzee iPSCs. (*A*) Plotting the average gene expression levels (TPM) in human and chimpanzee iPSC lines. Genes expressed significantly higher in human (191 genes) and chimp (146 gene) as well as the others (19,122) are shown. R indicates the Pearson’s R coefficient. (*B*) Expression levels (TPM) of pluripotency-related genes. (*C*) Expression levels of genes involved in TE restriction.

To compare gene expression patterns between human and chimpanzee iPSCs, mRNA-seq was performed in two female human iPSC lines and two female chimpanzee iPSC lines (supplementary table S1, Supplementary Material online), all of which were cultured in the same medium. The sequenced reads were mapped to both human and chimpanzee genomes (hg38 and panTro5, respectively), and the reads that were mappable to both genomes were used to estimate gene expression levels (see Materials and Methods). Using the data mapped onto the human genome (regardless of the species of samples) and the human gene annotation, gene expression levels were calculated, in terms of transcripts per million (TPM) (supplementary table S2, Supplementary Material online). Comparison of gene expression data (log-transformed) revealed a high similarity between the species (fig. 1*A*) When the transcriptomes were individually compared, Pearson’s *R* coefficients were 0.97–0.98 for intraspecies pairs and 0.96–0.97 for interspecies pairs. These data suggest that the gene expression pattern in iPSCs is highly conserved between human and chimpanzee.

Even under such transcriptomic similarity, 191 and 146 genes were identified as differentially expressed genes (DEGs), which were expressed to a higher degree in human and chimpanzee, respectively (≥2-fold, *q* < 0.05 by *t*-test with BH adjustment). Gene ontology (GO) analysis was conducted using Database for Annotation, Visualization and Integrated Discovery (Sherman et al. 2022) for these DEGs using a background gene list of 13,964 genes expressed at ≥1 TPM in iPSCs of either or both species. No GO term was enriched for the DEGs highly expressed in either species at an adjusted *P*-value of <0.05. Importantly, no pluripotency- or development-related GO term was enriched, and the gene expression levels of reprogramming factors, *POU5F1*, *SOX2*, *KLF4*, *MYC*, and *NANOG*, were similar between the two species (fig. 1*B*).

It has been reported that the expression levels of two TE-restricting genes, *PIWIL2* and *APOBEC3B*, are higher in human iPSCs than in chimpanzee iPSCs (Marchetto et al. 2013), and their human-specific expression has been proposed to cause a difference in the retrotransposition activity of L1HS and L1Pt (evolutionarily young LINE1 subfamilies in human and chimpanzee, respectively) between the species (Marchetto et al. 2013). However, in the iPSCs used in this study, *PIWIL2* was not highly expressed in either species (TPM = 0–0.48), while the higher expression of *APOBEC3B* in human was reproduced (fig. 1*C*). Analysis of published mRNA-seq data for human iPSCs and ESCs revealed low or no expression of *PIWIL2* in 7 of 9 cell lines analyzed (supplementary fig. S1, Supplementary Material online). Therefore, the upregulation of *PIWIL2* in human iPSCs seems to be specific to cell lines or culture conditions. It is of note that the cell lines with higher PIWIL2 expression, including those analyzed in Marchetto et al. (2013), were cultured in modified Tenneille Serum Replacer 1 medium. The PIWIL2 protein is involved in the production of 24–32-nucleotide small RNAs, called PIWI-interacting RNAs or piRNAs, in animal gonads (Czech et al. 2018). Consistent with the similar expression of *PIWIL2* in both species, small RNA-seq analysis disclosed a very limited number of piRNA-like RNAs in both species, with highly similar profiles (*R* = 0.90, supplementary fig. S1 and table S3, Supplementary Material online).

**Fig. 2.**
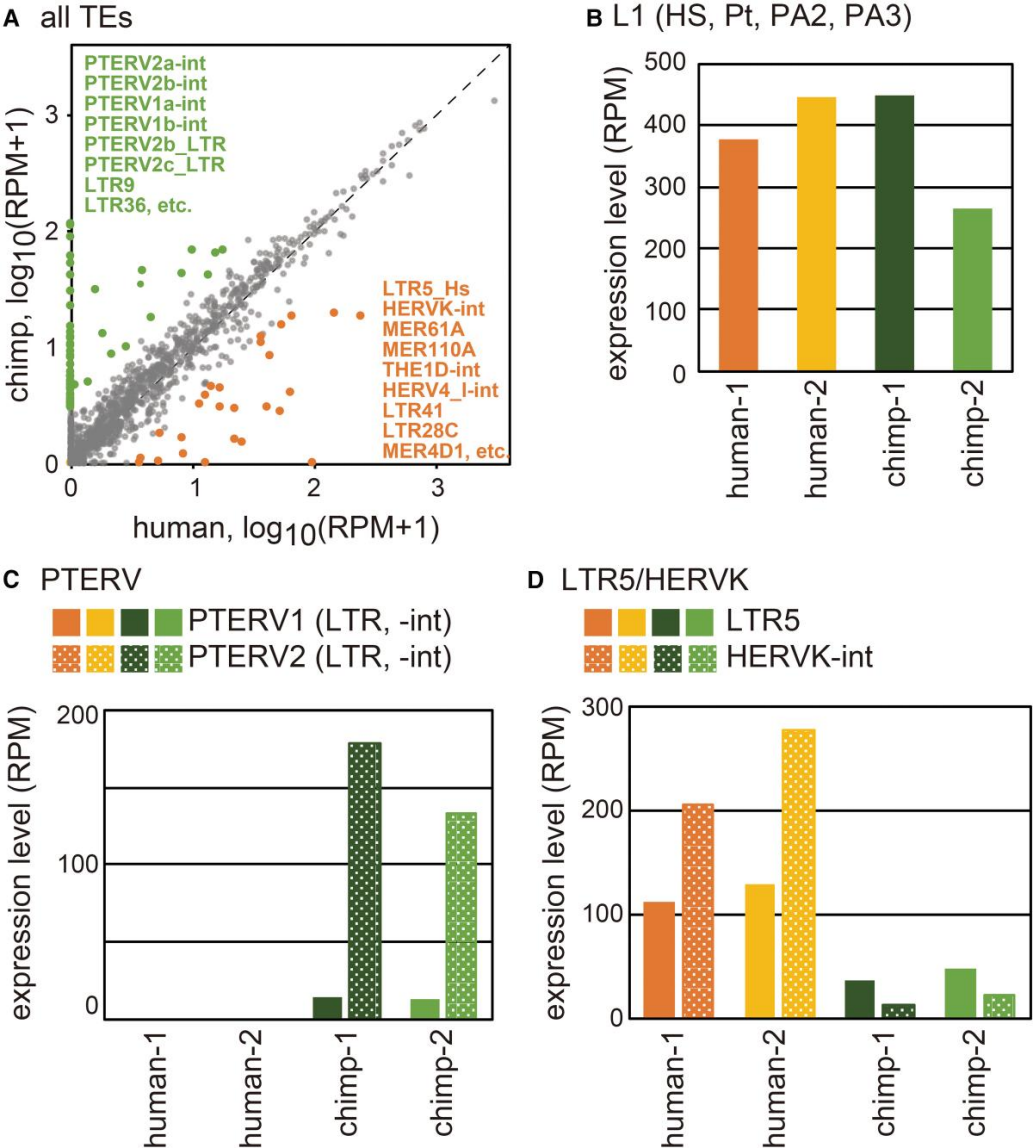
Comparison of TE expression between human and chimpanzee iPSCs. (*A*) Expression levels of TEs annotated in either or both of human and chimpanzee genomes are plotted. TEs showing a >3-fold difference are highlighted. (*B*) Sum of the expression levels of young L1 subfamilies (L1HS, L1Pt, L1PA2, and L1PA3). (*C*) Sum of the expression levels of chimpanzee-specific PTERVs (PTERV1*a*-int, PTERV1*b*-int, PTERV1*c*-int, PTERV1*d*-int, PTERV2*a*-int, PTERV2*b*-int, PTERV1*a*_LTR, PTERV1*c*_LTR, PTERV2*a*_LTR, PTERV2*b*_LTR, and PTERV2*c*_LTR). (*D*) Expression levels of LTR (LTR5, LTR5*A*, LTR5*B*, LTR_HS, and LTR5_Pt) and internal (HERVK-int) sequences.

Next, we calculated the expression level of each retrotransposon in each species using the mRNA-seq data. In this analysis, only the sense-strand expression was calculated (see Materials and Methods). Most of the retrotransposons were expressed at similar levels in the two species (*R* = 0.89, fig. 2*A* and supplementary table S4, Supplementary Material online). Although young L1 subfamilies have been reported to be more highly expressed in chimpanzee iPSCs (Marchetto et al. 2013), the cells used in this study did not show a significant difference between the species (fig. 2*B*). Some retrotransposons showed species-specific expression, most of which were species-specific families. For example, the PTERV family (PTERV1a, 1b, 1c, 1d, 2a, 2b, and 2c) is present only in the chimpanzee genome and showed chimpanzee-specific expression (fig. 2*C*). Among shared TEs, LTR5 and the associated internal HERVK (human endogenous retrovirus K) sequence were expressed to a greater degree in human iPSCs (fig. 2*D*).

### The Patterns of H3K4me3 and H3K27me3 in Human and Chimpanzee iPSCs

Histone modifications are important epigenetic modifications for the regulation of gene expression in a cell and/or later in development. In general, H3K4me3 is enriched in promoter regions of transcriptionally active or poised genes, whereas H3K27me3 is enriched in promoters and gene bodies of transcriptionally silenced genes. To compare these modifications between human and chimpanzee, ChIP-seq experiments were performed using the iPSCs. To avoid any bias introduced at the mapping steps, only sequence reads that were mappable to both human and chimpanzee genomes were used for downstream analysis. To make an interspecific comparison, human-genome mapping data of uniquely mapped read pairs were used for both species. First, we identified peaks for individual samples using ChIP and input reads. The length and ChIP enrichment of H3K4me3 peaks were comparable between the species, whereas the ChIP enrichment for H3K27me3 was slightly higher in chimpanzee samples (supplementary fig. S3, Supplementary Material online). This could be attributable to the smaller numbers of peaks in chimpanzee samples rather than a difference in the ChIP efficiency between the experiments. Thus, we concluded that the H3K4me3 and H3K27me3 profiles can be compared using these data.

**Fig. 3.**
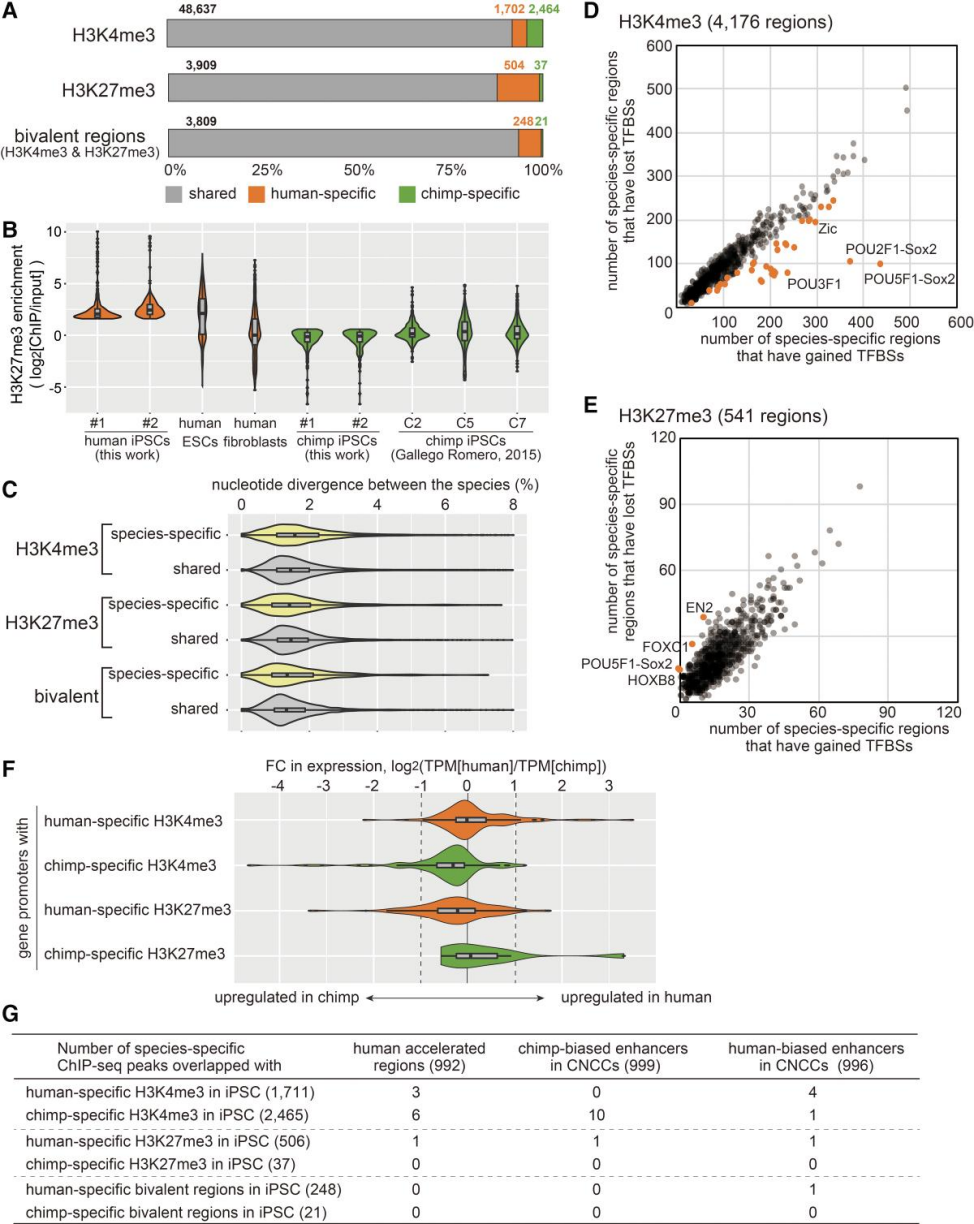
Histone modifications in human and chimpanzee iPSCs. (*A*) H3K4me3 and H3K27me3 ChIP-seq peak regions and bivalent peak regions in human and chimpanzee iPSCs. The numbers indicate numbers of shared, human-specific, and chimpanzee-specific regions, respectively. (*B*) Violin plots for the fold enrichments (log_2_[ChIP/input]) in the 504 human-specific H3K27me3 peaks calculated using the H3K27me3 ChIP-seq data of human-1, human-2, human ESCs (GSE29611), human fibroblasts (IMR90, GSE16256), chimp-1, chimp-2, and three other chimpanzee iPSCs (GSE69919). (*C*) Violin plots for the nucleotide divergence between the species in the H3K4me3, H3K27me3, and bivalent regions. (*D*) Loss and gain of transcription factor-binding sequence motifs in the species-specific H3K4me3 regions. Each plot represents a binding motif for respective TFs. The x-axis indicates the number of events where the species with H3K4me3 had a higher number of motifs than the other species. The y-axis indicates the number of events where the species with H3K4me3 had a lower number of motifs than the other species. Motifs with *P* < 0.001 (by χ^2^ test) are highlighted. (*E*) Loss and gain of transcription factor-binding sequence motifs in the species-specific H3K27me3 regions. (*F*) Violin plots for expression ratios (log_2_[human/chimpanzee]) in genes having species-specific histone-modified regions in their promoters. (*G*) Number of species-specific ChIP-seq peaks that overlapped with human accelerated regions (Prabhakar et al. 2006) and chimpanzee-biased and human-biased CNCC enhancers (Prescott et al. 2015). Numbers in parenthesis indicate total number.

To study the species-specificity of ChIP peaks, ChIP enrichment scores (the normalized number of ChIP reads over the normalized number of input reads) of all peaks identified in any one of the samples were calculated for all samples, and the averages of the respective species were compared (see Materials and Methods for criteria of species-specificity). Out of the 54,079 H3K4me3 peaks identified in human and/or chimpanzee, 2,006 and 2,810 were human-specific and chimpanzee-specific, respectively. To exclude regions unmappable uniquely, peaks where no read (input plus ChIP) was mapped in either species were discarded. To select regions with 1-to-1 orthology, orthologous regions of these shared or species-specific peaks were identified in the chimpanzee genome by liftOver, and the regions obtained were then “liftOvered” (carried over using liftOver) back to the human genome. We retained peaks that were appropriately liftOvered. This yielded a total of 52,803 peaks with validated orthology. Of these, 48,637 (92.1%), 1,702 (3.2%), and 2,464 (4.7%) H3K4me3 peaks were shared, human-specific, and chimpanzee-specific, respectively (supplementary tables S5 and S6, Supplementary Material online). Thus, the majority of peaks for this active chromatin mark were shared between the species, consistent with the transcriptomic conservation described above. On the other hand, the pattern of the H3K27me3 modification was more divergent: of the 4,450 H3K27me3 peaks with validated orthology, 504 (11.3%) and 37 (0.8%) were human- and chimpanzee-specific, respectively (supplementary tables S7 and S8, Supplementary Material online). Of note, human iPSCs had more H3K27me3-marked regions than chimpanzee iPSCs, despite that the ChIP enrichments for initially identified peaks were higher in chimpanzee (see above). The higher number of H3K27me3 peaks in human iPSCs is consistent with the previous report using different chimpanzee iPSC lines (Gallego Romero et al. 2015). It is formally possible that the differences in the H3K27me3 regions originated from differences in the epigenome in the respective source cells. Although the somatic cells of their origin are not available, analysis of published ChIP seq data for a human fibroblast cell line revealed low ChIP enrichments in the human iPSC-specific H3K27me3 regions, whereas human ESCs showed high enrichments in these regions (fig. 3*B*). These results suggest that the human-specific H3K27me3 regions do not represent “carryover” modifications that were inherited from the somatic source of the iPSCs; rather, they are likely specific to human cells of pluripotent state.

It has been suggested that cell lineage-specific genes are poised for expression in ESCs, by having both transcriptionally enhancing and repressing chromatin modifications, known as a bivalent state (Azuara et al. 2006; Bernstein et al. 2006; Hattori et al. 2013). To identify bivalent chromatin regions, the H3K4me3 and H3K27me3 peaks in individual cell lines were intersected. Comparison of these intersected regions (i.e., bivalent regions) between the species identified 248 (6.1%) and 21 (0.5%) bivalent regions that were specific to human and chimpanzee, respectively (supplementary tables S9 and S10, Supplementary Material online).

Since the H3K9me3 modifications in human and chimpanzee iPSCs were analyzed in a previous report (Ward et al. 2018), we compared the species-specific H3K4me3, H3K27me3, and bivalent peaks with H3K9me3 peaks. These peaks were merely (1.6% at most) overlapped to each other (supplementary fig. S4, Supplementary Material online), consistent with that of the H3K9me3 modification generally occurs in regions different from those enriched with H3K4me3 or H3K27me3.

### Genetic Origins of Species-specific Modifications

It is possible that the difference in the epigenetic marks is due to the evolutionary changes in the genomic sequence. Thus, we first compared the nucleotide divergences in regions having H3K4me3 and/or H3K27me3 in only one species (species-specific) and those in both species (shared). This revealed a similar trend of divergence (fig. 3*C*), indicating that these species specifically modified regions have not undergone accelerated evolution.

Consistent with this, the human accelerated regions (HARs) were underrepresented in species-specific peaks. The human genome contains regions that show significantly high conservation among non-human mammals, but a high frequency of nucleotide substitution in human, known as HARs. It has been suggested that HARs have a function in either increasing or decreasing enhancer activities involved in human-specific traits, including brain function (Pollard et al. 2006; Prabhakar et al. 2006, 2008; Lindblad-Toh et al. 2011; Capra et al. 2013). We intersected species-specific H3K4me3/H3K27me3 regions with the HARs (fig. 3*G*), which yielded very limited numbers of overlaps. This contrasted with the situation in human neural stem cells, wherein about 4% of HARs showed species-biased enhancer activities (Uebbing et al. 2021), hinting at a possibility that HARs are involved in human-specific changes in organogenesis, rather than in early development.

Next, we analyzed potential TFBSs in the species-specific regions using Find Individual Motif Occurrences (FIMO) (Grant et al. 2011) to identify species-specific loss and gain of TFBSs. This revealed that in species-specific H3K4me3 regions, the species showing the modification carried species-specific gain of 42 TFBS motifs (*P* < 0.001, χ^2^ test, orange in fig. 3*D*). Notably, these motifs included POU5F1-SOX2, POU2F1-SOX2, and ZIC, all of which are TFs acting in pluripotent cells. These results suggest that H3K4me3 regions can emerge during evolution, upon the occurrence of mutations that create binding sites for TFs working in the respective cells. The same analysis for the species-specific H3K27me3 regions disclosed the species-specific loss of TFBSs (fig. 3*E*). These TFBSs again included POU5F1-SOX2, suggesting that POU5F1 and SOX2 are important factors that dictate the species-specific epigenome of iPSCs in human and chimpanzee.

### Species-specific Modifications are Correlated With the Gene Expression Difference in iPSCs

As many human- or chimpanzee-specific H3K4me3 or H3K27me3 regions overlapped with promoters (within 2-kb upstream and 0.5-kb downstream from a transcription start site), we compared the expression levels of the associated genes. Consistent with the roles of H3K4me3 and H3K27me3 in gene regulation, genes with human-specific H3K4me3 or chimpanzee-specific H3K27me3 displayed upregulation in human iPSCs, as compared to chimpanzee iPSCs, while genes with chimpanzee-specific H3K4me3 or human-specific H3K27me3 displayed upregulation in chimpanzee iPSCs (fig. 3*F*).

The species-specific peaks outside gene promoters may be linked to the regulation of the enhancer activity. To assume candidate genes under their regulation, the nearest genes to the human- and chimpanzee-specific peaks were identified, following which GO enrichment analysis was carried out using Genomic Regions Enrichment of Annotations Tool (McLean et al. 2010). However, no GO term was enriched in any category (human- or chimpanzee-specific H3K4me3, H3K27me3, or bivalent regions).

### Interspecific Epigenetic Differences in CNCCs Appeared Late in Differentiation

Species-specific enhancer activities and histone modification patterns have been revealed in human and chimpanzee CNCCs that were derived from iPSCs by means of in vitro differentiation (Prescott et al. 2015). We compared chimpanzee-biased enhancers (more active in chimpanzee) in CNCCs with chimpanzee-specific H3K4me3 and human-specific H3K27me3, which revealed that only a limited number of these regions were overlapped (fig. 3*G*). Likewise, human-biased enhancers did not overlap well with human-specific H3K4me3 or chimpanzee-specific H3K27me3. Therefore, it is likely that most of the epigenetic differences in CNCC enhancers appeared late during differentiation.

### Species-specific LTR5 Insertions Resulted in H3K4me3 Modifications Associated with Gene Expression Changes

**Fig. 4.**
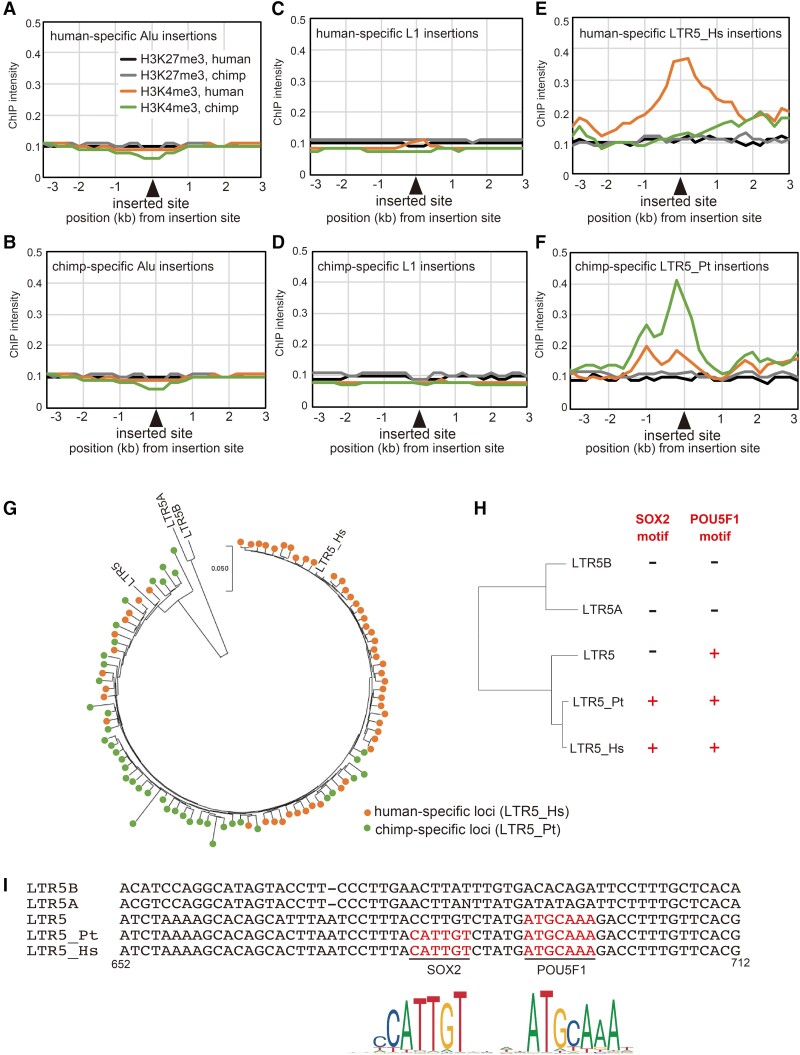
Epigenetic states around species-specific TE insertions. (*A*–*F*) ChIP-seq read intensities around 2,896 human-specific Alu (*A*), 1,050 chimpanzee-specific Alu (*B*), 983 human-specific L1 (*C*), 1,298 chimpanzee-specific L1 (*D*), 58 human-specific LTR5 (*E*), and 42 chimpanzee-specific LTR5 (*F*) insertions. For each species, ChIP-seq intensities were calculated as an average for all species-specific insertions in the two cell lines. (*G*) An NJ tree of species-specific LTR5 insertions. (*H*) An NJ tree of the consensus sequences of LTR5, LTR5*A*, LTR5*B*, LTR5_Hs, and LTR5_Pt. The presence (+) or absence (−) of SOX2 and POU5F1 motifs are indicated on the right. (*I*) Sequence alignment of LTR5 subfamilies in regions covering the POU5F1-SOX2 dual motif (positions 652 to 712 in LTR5_Hs). SequenceLogo representations of SOX2 and POU5F1 are shown at the bottom.

Retrotransposition of retrotransposons in a species generates the interspecific genomic difference and potentially the epigenomic difference as well. Therefore, we analyzed the ChIP-seq data for regions flanking species-specific insertions of retrotransposons (see Materials and Methods for identification of species-specific retrotransposon insertions and ChIP analysis). Insertions of Alu and L1 did not induce a change in H3K4me3 or H3K27me3 (fig. 4*A*–*D*). However, as reported recently (Zhuo et al. 2020), human-specific LTR5 insertions induced H3K4me3 in flanking regions of 2 kb on both sides (fig. 4*E*). Chimpanzee-specific LTR5 insertions also induced H3K4me3 (fig. 4*F*). In human, three LTR5 subfamilies are present, LTR5A, LTR5B, and LTR5_Hs. Phylogenetic analysis of human-specific LTR5 insertions (regardless of their H3K4me3 modification) showed that all insertions belong to LTR5_Hs (fig. 4*G*). In addition to a binding site for POU5F1 reported previously (Grow et al. 2015), we found a SOX2 binding motif neighboring the POU5F1 site, thus creating a POU5F1-SOX2 dual binding motif in LTR5_Hs (fig. 4*H* and *I*). In chimpanzee, only one subfamily has been reported (LTR5). Phylogenetic analysis of chimpanzee-specific LTR5 insertions revealed that they are similar to the sequence of LTR5_Hs (fig. 4*G*). These copies form an active subfamily, and their consensus sequence is more similar to LTR5_Hs than to LTR5 (fig. 4*H*). We designated this subfamily as LTR5_Pt (Pt stands for *Pan troglodytes*). Importantly, the consensus sequence of LTR5_Pt also carries a POU5F1-SOX2 dual motif (fig. 4*H* and *I*). Therefore, it is conceivable that species-specific insertion of LTR5_Hs or LTR5_Pt generates a new POU5F1-SOX2 motif, which serves as a nucleation site of the H3K4me3 modification.

**Fig. 5.**
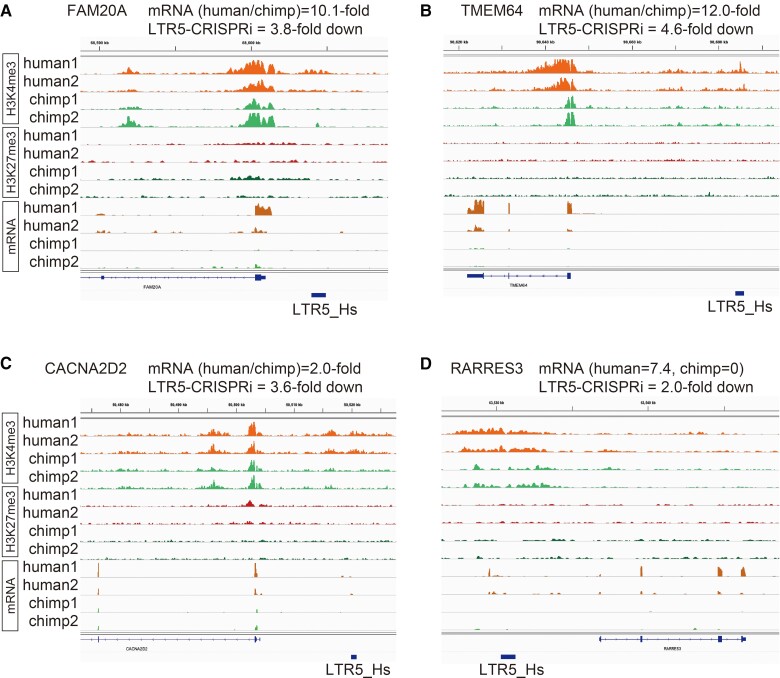
Examples of LTR5_Hs-induced gene upregulation in human iPSCs. H3K4me3 and H3K27me3 ChIP-seq and mRNA-seq data are shown for the promoter proximal regions of *FAM20A* (*A*), *TMEM64* (*B*), *CACNA2D2* (*C*), and *RARRES3* (*D*). The locations of human-specific LTR5_Hs insertions are shown on the bottom. Gene expression differences (human average vs. chimpanzee average) are shown above the IGV snapshots. The levels of downregulation in CRISPRi experiments targeting LTR5_Hs (Fuentes et al. 2018) are also shown.

Moreover, some of the species-specific LTR5_Hs or LTR5_Pt insertions located close to genes were associated with differences in gene expression levels between the species. For example, an LTR5_Hs copy is inserted upstream of *FAM20A*, which generated human-specific H3K4me3 (fig. 5*A*), the expression level of *FAM20A* was 10-fold higher in human iPSCs, suggesting that the LTR5_Hs insertion serves as an enhancer. Consistent with this possibility, it has been reported that *FAM20A* was downregulated by 3.8-fold when a bulk of LTR5_Hs copies were altered to have a repressive modification in human embryonal carcinoma cells, using the CRISPRi system (Fuentes et al. 2018). Similarly, when LTR5_Hs copies were inserted close to *TMEM64* (fig. 5*B*), *CACNA2D2* (fig. 5*C*), *RARRES3* (fig. 5*D*), *SEMA4A* (supplementary fig. S5*A*, Supplementary Material online), and *MMP24* (supplementary fig. S5*B*, Supplementary Material online), these genes were expressed to a greater extent in human iPSCs (supplementary table S2, Supplementary Material online) and downregulated upon carrying out LTR5_Hs-CRISPRi (Fuentes et al. 2018). In case of an insertion upstream of *RARRES3*, the LTR5_Hs generated an alternative transcription start site, and the resulting transcript was spliced to the first or second exon of the gene (supplementary fig. S5*C*, Supplementary Material online). Other examples did not show such fusion transcripts and likely served as enhancers. When two instances of LTR5_Pt were inserted close to *PADI2* and *FAM149B*, it made H3K4me3 regions associated with chimpanzee-biased expression of these genes (supplementary table S2, Supplementary Material online). Altogether, these results argue in favor of the fact that species-specific LTR5 insertions can generate gene expression differences by means of their enhancer or promoter activities in undifferentiated cells during embryonic development.

Whereas we found the POU5F1-SOX2 motif in both of LTR5_Hs and LTR5_Pt, it has been reported that the LTR5_Hs subfamily emerged in the human genome after the divergence of human and chimpanzee (Buzdin et al. 2003). To study when LTR5 copies with the POU5F1-SOX2 motif emerged, we analyzed 635 LTR5_Hs copies in the human genome. Their orthologous regions in the chimpanzee genome were identified, revealing that 300 LTR5_Hs copies had orthologous LTR5 copies in chimpanzee (see Materials and Methods). Of these 300 LTR5_Hs copies, 265 copies carried the POU5F1-SOX2 motif. Of the chimpanzee copies orthologous to these 265 copies, 257 (97%) carried the POU5F1-SOX2 motif, suggesting strongly that the origin of the motif in these LTR5 copies dated back to the common ancestor of human and chimpanzee. Moreover, of these 257 copies, 205 copies had orthologs in the gorilla genome (gorGor6) with 190 copies carrying the motif. Therefore, LTR5 likely acquired the POU5F1-SOX2 motif before the divergence of human, chimp, and gorilla. Afterwards, such LTR5 subfamilies have proliferated in the respective genomes and likely have generated different patterns of gene expression.

## Discussion

According to the original definition, “epigenetics” refers to a type of phenotypic change that is heritable through cell division but does not involve a DNA mutation. Such epigenetic phenomena involve chemical modifications of DNA and histones in nucleosomes, such as H3K4me3 and H3K27me3 for gene activation and repression, respectively. Different types of cells in the same individual show different overall epigenetic states, called epigenomes, which are acquired during developmental differentiation, although the exact program that governs epigenome dynamics remains unknown. To understand the mechanisms of interspecific phenotypic differences, it is important to understand interspecific epigenomic differences in tissues and cells, how such differences emerge during development, and how genetic and epigenetic changes are associated.

The generation of iPSCs in human and non-human primates and in vitro differentiation methods into specific cells or organoids have offered a great opportunity to study evolutionary changes in the programmed developmental dynamics of the transcriptome and epigenome. Thus, in the present study, a comparative analysis of the starting iPSCs of human and non-human primates was performed in terms of the transcriptome and epigenome by performing mRNA-seq, small RNA-seq, and ChIP-seq of sex- and age-matched human and chimpanzee iPSCs. Our results showed that a vast majority of the gene expression and histone modification patterns were conserved between the two species (figs. 1 and 3). This is consistent with the fact that these cells were seemingly similar in morphology, stem cell characteristics, and ability to differentiate into three germ layers. With these high similarities, iPSCs can be used to delineate the trajectories of epigenome dynamics during differentiation, which would elucidate how species-specific and conserved epigenetic states in pluripotent cells will change or remain during development and how such differences are involved in transcriptomic and phenotypic divergence.

On the other hand, we identified some differences between iPSCs, which seem to depend on the underlying genome sequence. In the present study, we identified 4,176 species-specific H3K4me3 and 541 species-specific H3K27me3 regions in human and chimpanzee genomes (fig. 3*A*). Species-specific H3K4me3 and H3K27me3 regions showed no acceleration of mutations in either species. However, we found that about 10% of these regions had nucleotide substitutions that resulted in POU5F1-SOX2 binding motifs that were present only in the species with H3K4me3 (fig. 3*D*). POU5F1 (also called OCT4) and SOX2 are TFs that are active in iPSCs; indeed, both human and chimpanzee iPSCs expressed these factors at similar levels (fig. 1). This is along the same lines of a previous finding that sequence changes in binding motifs for cell-type-specific TFs result in interspecific DNA methylation differences in the cells that express the respective TFs (Fukuda et al. 2017). Together, these results suggest that evolutionary gains of TF binding by sequence alterations generate chromatin environments for gene activation. This seems to be contrary to the original definition of epigenetics, which does not involve the alteration of the DNA sequence. At present, it is known that epigenetic modifications are used to decode genetic information spatiotemporally, and the program when and where a particular genomic region is decoded is likely to be dictated by the combinations of TFs present in a cell. Because such sequence changes in TFBSs induce local epigenetic changes in confined types of cells, while maintaining the epigenome globally, these genetic changes play a role in evolutionary changes in the epigenetic program of embryonic development.

The present study also revealed that gains of POU5F1-SOX2 motifs occurred not only by means of base substitution but also by retrotransposition of LTR5_Hs and LTR5_Pt (fig. 4*H* and *I*). This is consistent with a recent report that LTR5_Hs insertions create human-specific H3K4me3 peaks in human iPSCs (Zhuo et al. 2020). In the present study, we revealed that LTR5_Hs and LTR5_Pt carry a SOX2 binding motif (nucleotide positions 681–686), in addition to the previously identified POU5F1 motif (positions 692–698) (Glinsky 2015; Grow et al. 2015), which together form a POU5F1-SOX2 dual binding motif. Consistently, it was recently reported that the ChIP-seq data of both POU5F1 and SOX2 showed a peak in the region encompassing this motif in LTR5_Hs in human iPSCs (Monde et al. 2022) and human ESCs (Zhang et al. 2022). Owing to the sequence motif, retrotransposed LTR5_Hs and LTR5_Pt copies bind to POU5F1 and SOX2 and gain active histone marks, which underlie the species-specific active chromatin environment and activation of nearby genes in iPSCs.

Despite the similar levels of expression of POU5F1 and SOX2 (fig. 1*B*), the expression of the LTR5 family was higher in human than in chimpanzee (fig. 2*A*). This may be explained by the binding-site difference(s) for other TFs between LTR5_Hs and LTR5_Pt. For example, LTR5_Hs, but not LTR5_Pt, carries a binding site for ETV1 (positions 195–208), which is highly expressed in iPSCs (at a level comparable to SOX2 and NANOG) and known to have a function to activate transcription (Hollenhorst et al. 2011). In any event, it is likely that LTR5_Hs can be retrotransposed in cells expressing POU5F1 and SOX2, such as pluripotent cells in the blastocyst and epiblast, and primordial germ cells. Supporting this idea is a recent report that endogenous LTR5_Hs/HERVK copies can be retrotransposed in human iPSCs (Monde et al. 2022). Since embryonic pluripotent cells and primordial germ cells have the potential to become gametes later in development, retrotransposition of LTR5_Hs/HERVK in these cells can support successful transmission of new copies to the subsequent generation.

In contrast to the results that similar numbers of human- and chimpanzee-specific H3K4me3 regions were identified, there were 13 times more human-specific H3K27me3 regions than chimpanzee-specific regions. These human-specific H3K27me3 regions are frequently marked with H3K4me3 in both species, generating human-specific bivalent regions. The species-specific H3K27me3 regions did not show an accelerated rate of sequence substitutions (fig. 3*C* and *G*) and were not frequently associated with loss of TFBSs (fig. 3*E*). This suggests that, in comparison to active chromatin, the establishment and maintenance of repressive chromatin are more sequence-independent and thus *epigenetic*. For example, the different activities of histone methylases and/or demethylases may underlie evolutionary changes in the locations of repressive chromatin. It is also possible that interspecific differences in the chromatin environment of the nuclear space, which could be influenced by the cell’s past and current experiences, are involved in the generation of epigenetic differences.

## Materials and Methods

### Human and Chimpanzee iPSCs

Two human iPSC lines, 409 B2 (derived from a 36-year-old female; designated as human-1) and Nips B2 (derived from a 43-year-old female; designated as human-2), were obtained from RIKEN Cell Bank, Japan. Two chimpanzee (*Pan troglodytes*) iPSC lines, 0138F-1 (derived from a 39-year-old female; designated as chimp-1) and 0274F-2 (derived from a 39-year-old female; designated as chimp-2), were established previously (Kitajima et al. 2020). The detail information was shown in supplementary table S1, Supplementary Material online. The iPSCs of both species were cultured on iMatrix-511 (Takarabio, Kusatsu, Japan)-coated 60 mm cell culture plates in StemFit® (Ajinomoto, Tokyo, Japan) and incubated at 37 °C in 5% CO_2_.

### RNA Extraction, Library Preparation, and Sequencing

Total cellular RNAs were extracted using Isogen (Toyobo, Osaka, Japan) and Direct-Zol™ RNA (Zymo Research, Irvine, USA). After quality check using Agilent 2100 Bioanalyzer (Agilent Technologies, Santa Clara, USA), polyA-containing mRNAs were purified using NEBNext® Poly(A) mRNA Magnetic Isolation Module (New England Biolabs, Ipswich, USA) and used for the preparation of indexed mRNA-seq libraries using NEBNext® Ultra Directional RNA Library Prep Kit for Illumina (New England Biolabs). The libraries were sequenced on HiSeq X™ Ten (Illumina, San Diego, USA), in the 150-bp paired-end mode. For each sample, 8–85 million read pairs were obtained.

Indexed small RNA-seq libraries were prepared from total RNAs, using NEBNext® Small RNA Library Prep Set (New England Biolabs). After library amplification using PCR, the products were separated using 6% polyacrylamide gel electrophoresis, and a gel region corresponding to DNA sizes with 15–40 bp insertion was cut for DNA extraction. The libraries were sequenced on HiSeq1500 (Illumina), in the 50-bp single-end mode. For each sample, 11–16 million reads were obtained.

### mRNA-seq Data Analysis

Trim Galore! (https://www.bioinformatics.babraham.ac.uk/projects/trim_galore) was used to remove the adapter sequences and 3′ nucleotides with low-quality scores (*Q* < 20). The retained reads were mapped to both the human (hg38) and chimpanzee (panTro5) reference genomes, using Hisat2 (Kim et al. 2019). To prevent biases in expression-level estimates due to the relatively poor annotation of the chimpanzee genome, and genomic deletions and insertions between the species, we first mapped the reads onto panTro5, regardless of the species of mRNA origin, following which only the mapped reads were then mapped onto hg38. In all samples, more than 79% reads were mapped onto both genomes. After removing duplicated reads by using SAMtools (Danecek et al. 2021), the mapping data on hg38 was used to analyze gene expression levels using StringTie (Pertea et al. 2015) with the human genome annotation and the –fr option, giving TPM for each gene. Despite a large difference in read number between human-1 (8 million), human-2 (84 million), chimp-1 (31 million), and chimp-2 (23 million), they showed high concordance in the overall transcriptome (see main text).

To identify DEGs, the average TPM values were compared between species by student *t*-test, and the fold change was calculated as (TPM_human_ + 1)/(TPM_chimp_ + 1). Genes were selected as DEGs if the Benjamini-Hochberg-adjusted *P*-value was <0.05 and a fold change was either ≥2 or ≤0.5.

To analyze the TE expression, human RNA-seq reads were mapped onto hg38, and chimpanzee RNA-seq reads were mapped onto panTro5, using Hisat2, allowing multiple hits but outputting a randomly selected one from the candidate regions. We counted second reads (representing sense-strand sequences in more 5′ proximal regions than their counterpart reads) that were aligned in sense orientation with the RepeatMasker Track downloaded from the UCSC table browser (Karolchik et al. 2004). The expression level of TE was normalized as RPM (reads per million reads mappable to the genome at least once).

### Small RNA-seq Data Analysis

Cutadapt (DOI: 10.14806/ej.17.1.200) was used to remove the adapter sequences, following which the retained reads of 24–35 bp in length (corresponding to small RNAs of 24–35 nucleotides, which is the range for typical piRNAs) were mapped to the respective reference genomes (hg38 or panTro5) using Hisat2 with options, –score-min L,0,0 (allowing no mismatch) and -a (reporting all mapped regions). Reads that originated from TE regions in the RepeatMasker track were counted and normalized as RPM. If a read was mapped to multiple candidate regions, all regions were used for read counting, using a weighted number, 1/*n*, where *n* is the number of mapped regions.

### Preparation of Native Chromatins and ChIP

Pellets of ∼1 × 10^6^ cells were re-suspended in 50 µL of buffer I (300 mM sucrose, 60 mM KCl, 15 mM NaCl, 5 mM MgCl_2_, 0.1 mM EGTA, 15 mM Tris-HCl pH 7.5, 5 mM sodium butyrate, and 0.5 mM DTT), following which an equal volume of buffer II (300 mM sucrose, 60 mM KCl, 15 mM NaCl, 5 mM MgCl_2_, 0.1 mM EGTA, 15 mM Tris-HCl pH 7.5, 5 mM sodium butyrate, and 0.5 mM DTT) was added. After incubation on ice for 10 min, the cell suspensions were layered over 900 µL of buffer III (1.2 M sucrose, 60 mM KCl, 15 mM NaCl, 5 mM MgCl_2_, 0.1 mM EGTA, 15 mM Tris-HCl pH 7.5, 5 mM sodium butyrate, and 0.5 mM DTT). Nuclei were collected by means of centrifugation at 8,000 rpm at 4 °C for 20 min. Nuclear pellets were washed in 200 µL of MNase digestion buffer (320 mM sucrose, 50 mM Tris-HCl pH 7.5, 4 mM MgCl_2_, 1 mM CaCl_2_, and 5 mM sodium butyrate) and then re-suspended in 20 µL of MNase digestion buffer. For ChIP experiments against H3K4me3, chromatins were incubated with micrococcal nuclease (MNase) (Takarabio) at a final concentration of 4 mU/µL, at 37 °C for 20 min, in a 40-µL reaction mixture. Chromatin for ChIP experiments against H3K27me3 was incubated with MNase (New England Biolabs), at a final concentration of 0.27 gel units/µL, at 37 °C for 10 min. Digestion was stopped by adding 2 µL of 0.5 M EDTA on ice. Two hundred microliters of incubation buffer (50 mM NaCl, 5 mM EDTA, 0.01% NP-40, 20 mM sodium butyrate, and 20 mM Tris–HCl pH 7.5) was then added to the samples and they were centrifuged at 13,000 rpm, at 4 °C for 10 min. The supernatants were collected as chromatin samples, and an aliquot of 100 µL was incubated with a rabbit polyclonal antibody against H3K4me3 (Merck Millipore Darmstadt, Germany) (catalog number 07-473, 2 µL) or a rabbit monoclonal antibody against H3K27me3 (Cell Signaling Technology, Danvers, USA) (catalog number 9733, 2 µL), at 4 °C for 1 h, and captured using anti-IgG antibody-conjugated Dynabeads® M-280 (Thermo Fisher Scientific). The immune complexes were sequentially washed in 400 µL of wash buffer A (75 mM NaCl, 10 mM EDTA, 50 mM Tris-HCl pH 7.5, 0.01% NP-40, and 5 mM sodium butyrate), 400 µL of wash buffer B (100 mM NaCl, 10 mM EDTA, 50 mM Tris-HCl pH 7.5, 0.01% NP-40, and 5 mM sodium butyrate) and 400 µL of wash buffer C (175 mM NaCl, 10 mM EDTA, 50 mM Tris-HCl pH 7.5, 0.01% NP-40, and 5 mM sodium butyrate). The beads were incubated in 200 µL of lysis buffer (400 mM NaCl, 10 mM EDTA, 0.005% SDS, 20 mM Tris-HCl pH 8.0, and 0.1 mg/ml proteinase K) at 56 °C for 1 h, and the DNA in the supernatant was obtained by means of phenol/chloroform extraction and ethanol precipitation.

### ChIP-seq Library Preparation and Data Analysis

Using the ChIP DNAs obtained, sequencing libraries were generated using NEBNext® ChIP-Seq Library Prep Master Mix Set for Illumina (New England Biolabs). The libraries were sequenced on HiSeq X™ Ten (Illumina), in the 150-bp paired-end mode. For each sample, 18–38 million read pairs were obtained. The sequence reads were processed using Trim Galore!, as described in the mRNA-seq analysis section. The retained reads were mapped to both the reference sequences (hg38 and panTro5), using Hisat2 with options, –no-discordant and –no-spliced-alignment. Only the reads that were mapped to both genomes were retained and used for the downstream analysis. PCR duplicates were removed by using SAMtools. ChIP-seq peaks were identified along the hg38 genome using the *peakcall* function of MACS2 (Zhang et al. 2008), with options, –broad, -f BAMPE, and -g hs. Bam files for ChIP and input were used to identify peaks.

### Identification of Species-specific ChIP-seq Peaks

After peak identification, the number of reads (ChIP and input) that overlapped with the respective peaks was counted for each sample using the *coverage* function of BEDTools (Quinlan and Hall 2010) and divided by the total number of genome-mapped reads. Following that, for each peak, the value calculated for the ChIP sample was divided by that for the input sample, thus resulting in an enrichment score. In this step, regions where no read was uniquely mapped in either species were discarded. To check the 1-to-1 orthology of the peak regions, liftOver (a tool available in the UCSC genome browser) was used for the peaks identified in hg38 to find their orthologous regions in panTro5. Regions that were deleted or duplicated in panTro5 were discarded, and the retained regions were analyzed by liftOver back to hg38. If the identified regions were same as their original regions, we regarded these regions as validated orthology and retained them for downstream analyses. The human-specific peaks were ones where the average of enrichment scores in human samples was ≥3 and the average of enrichment scores in chimpanzee samples was <1.5. Chimpanzee-specific peaks were identified in the same way.

### Sequence Comparison for Species-specific Peaks

For human-specific, chimpanzee-specific, and shared peaks, the sequence identity between the orthologous regions was analyzed using Basic Local Alignment Search Tool (Altschul et al. 1990). Both human and chimpanzee sequences in the respective peaks were analyzed using FIMO (Grant et al. 2011) for the presence of TF-binding motifs, using position frequency matrices obtained from JASPAR (https://jaspar.genereg.net).

### Identification of Species-specific Retrotransposon Insertions and Analysis of Their Flanking Regions

The RepeatMasker Track of the panTro5 genome assembly was converted to hg38 coordinates (i.e., counterpart genome) using liftOver and vice versa. The repeats that lacked orthologous regions were collected as candidates for species-specific insertions. Their 1-kb flanking regions on both sides were then converted to their counterpart genome using liftOver. Species-specific insertions were selected if their flanking regions were present in the counterpart genome in tandem, and were appropriately ‘liftOvered’ (carried over using liftOver) back to the original genome. This identified 5,422 human-specific and 3,625 chimp-specific retrotransposon insertions, with the majority being Alu (∼70%) and L1 (∼20%) insertions. For each specific insertion in each species, 30 flanking regions of 200-bp (15 upstream and 15 downstream regions) were converted to the counterpart genome (The 1-to-1 orthology for these regions was validated by liftOver back to the original genome). Following that, using the *coverage* function of BEDTools (Quinlan and Hall 2010), human ChIP-seq reads were counted for each 200-bp region arrayed in the hg38 coordinate, and chimpanzee ChIP-seq reads were counted for each 200-bp region arrayed in the panTro5 coordinate. Read counts were normalized to the total mapped reads and the averages of the respective species were compared.

### Analysis of Motif Sequences in LTR5

Human LTR5_Hs copies that are ≥500 bp in length were extracted from the RepeatMasker Track of hg38, and their orthologous regions in the chimpanzee genome (panTro5) were identified by liftOver, which was then intersected by regions annotated as LTR5 in panTro5. This yielded 300 LTR5 copies shared between human and chimpanzee, and their orthology was confirmed by liftOver back to hg38. Their sequences in hg38 and panTro5 were analyzed using FIMO to check whether they carry the POU5F1-SOX2 motif. For the 265 LTR5 copies carrying the motif in both human and chimpanzee, their orthologous regions in gorilla (gorGor6) were identified by liftOver, and intersected with the RepeatMasker Track of gorGor6 to confirm if they were annotated as LTR5. Their sequences were analyzed using FIMO.

## Supplementary Material

msac208_Supplementary_Data

## Data Availability

The mRNA-seq, small RNA-seq, and ChIP-seq data have been deposited to Gene Expression Omnibus under the accession number, GSE201298.
