# Supplementary material for: Sequence Divergence and Retrotransposon Insertion Underlie Interspecific Epigenetic Differences in Primates
Source: Mol Biol Evol. 2022 Oct 11;39(10):msac208. doi: 10.1093/molbev/msac208 (PMC9577543; doi:10.1093/molbev/msac208)
Supplement: msac208_Supplementary_Data [file msac208_supplementary_data.zip › Hirata_Suplementary_FigS1_to_S5.pdf]

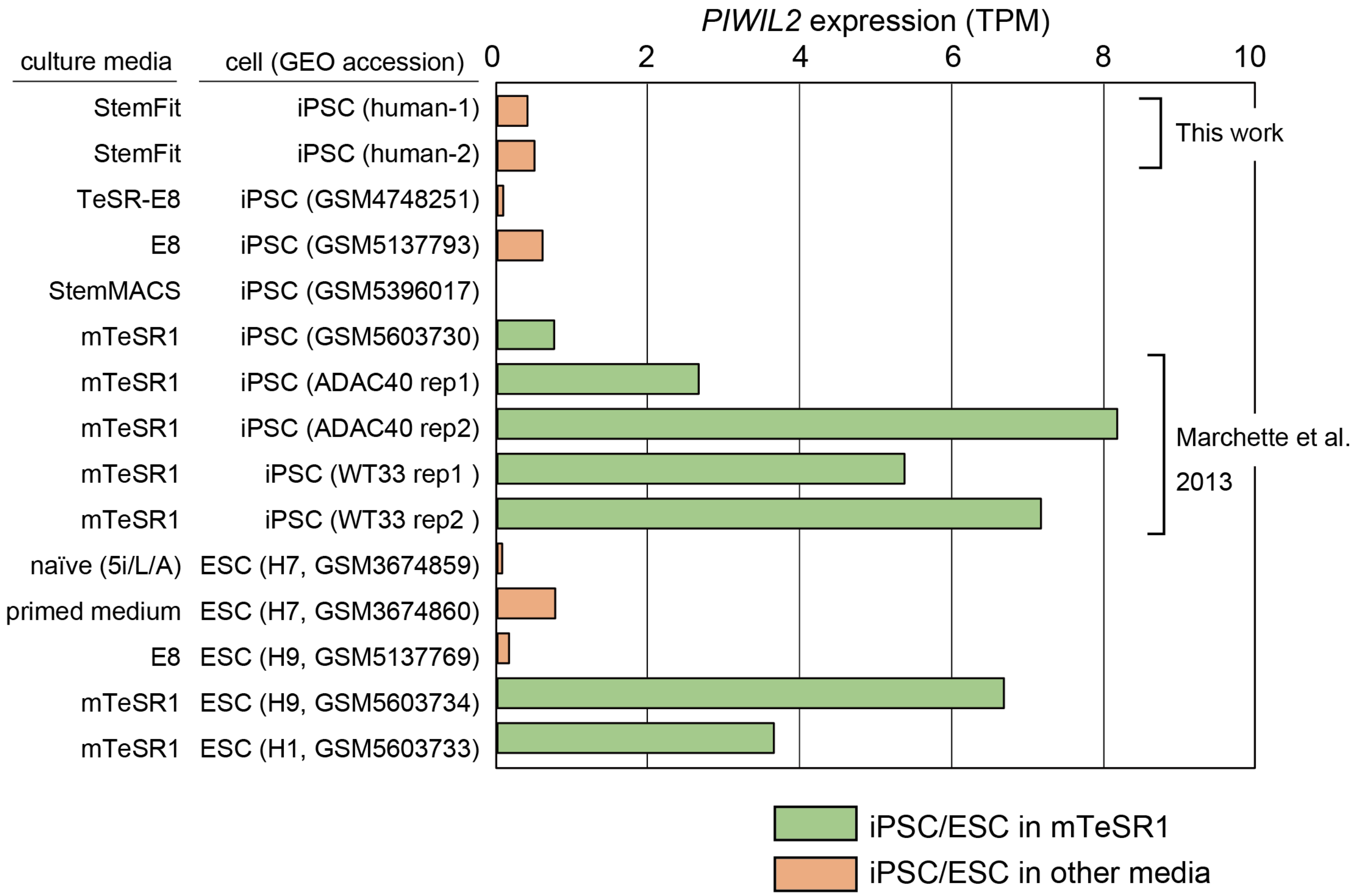

Figure S1. PWIL2 expression level in various human iPSCs and ESCs

Published mRNA-seq reads were downloaded from GEO and the gene expression levels were determined by using Hisat2 and StringTie. Expression in mTeSR1 media and others are shown in green and orange, respectively, to show that culture in mTeSR1 seems to be associated with higher *PIWIL2* expression.

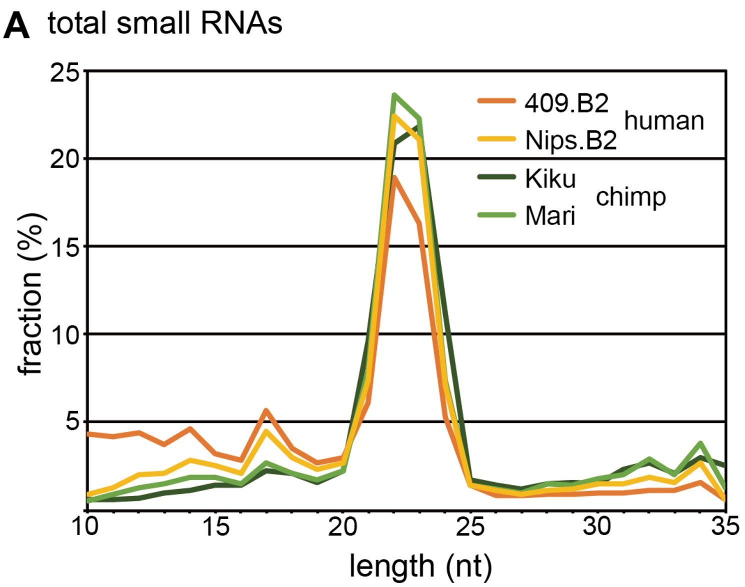

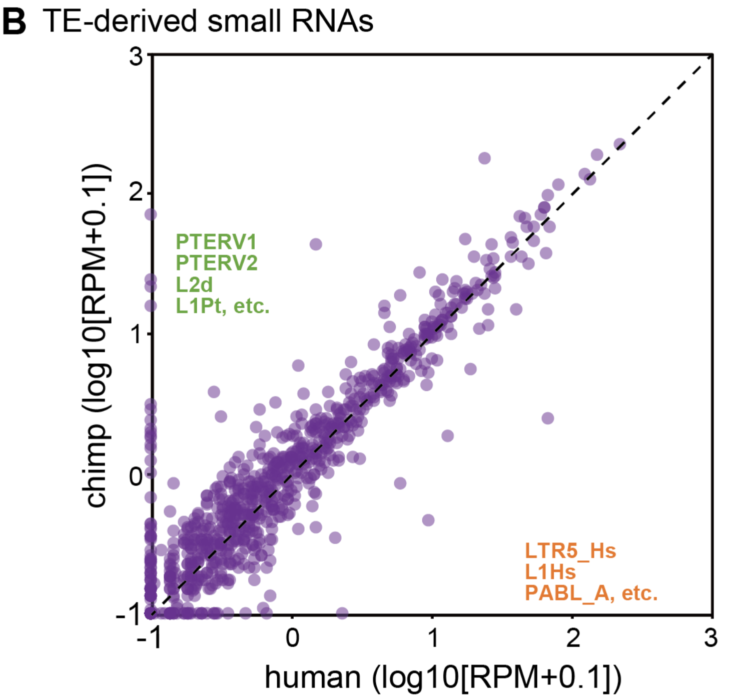

Figure S2. small RNA profiles in human and chimpanzee iPSCs.

(A) Length profiles of RNA sequences (rRNA and snRNAs were removed). Most of reads were from miRNAs of 21-23 nt, and >24-nt RNAs were very limited, indicating little, if any, production of piRNAs in iPSCs.

(B) Log10 expression levels of small RNAs (24 to 35 nt long) derived from TEs. Small RNAs were mapped to the respective reference genomes, and reads mapped within TEs were counted for each TE families. Examples of TEs whose small RNAs were specifically expressed in human and chimpanzee are shown as orange and green, respectively. Most of them are species-specific families.

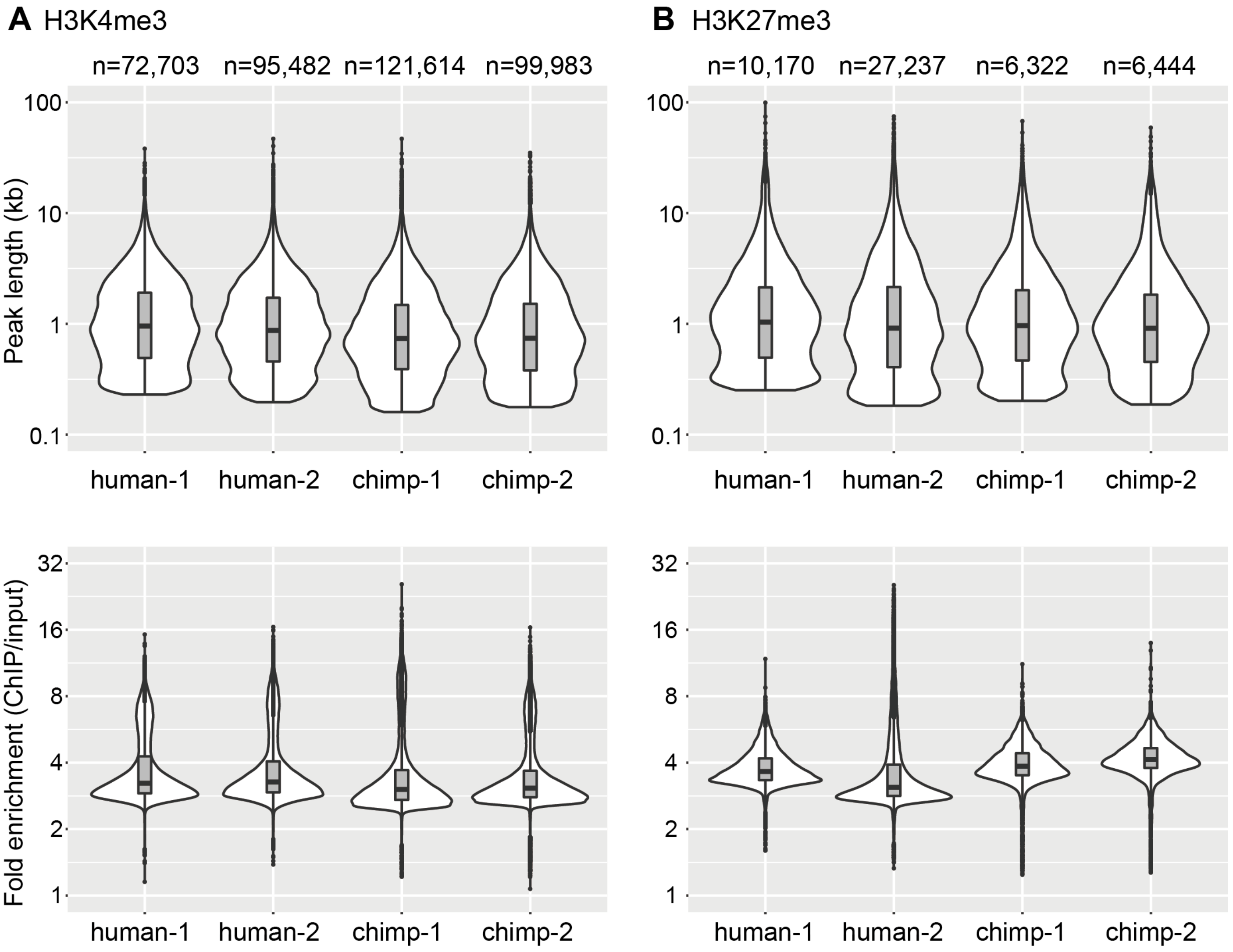

Figure S3. Quality comparison of ChIP-seq peaks identified by MACS2

Statistics of the length (upper) and fold enrichent (lower) of H3K4me3 (A) and H3K27me3 (B) ChIP-seq peaks initially identified by MACS2, using the respecitive ChIP and input sequencing reads for each sample.
The numbers of the peaks were indicated on the top. Note that these peaks were identified individually. For the respecitive modifications, detected peaks were merged and anlyzed for species-specificity (see the main text).

		total	overlapped with H3K9me3 peaks		
			human-specifc	chimp-specific	shared
H3K4me3	human-specifc	1,702	0	28	2
	chimp-specific	2,463	1	7	3
	shared	48,637	16	113	33
H3K27me3	human-specifc	504	0	0	2
	chimp-specific	37	0	0	0
	shared	3,909	0	5	4
bivalent	human-specifc	248	0	0	1
	chimp-specific	21	0	0	0
	shared	3,809	0	1	2

Figure S4. Number of peaks overlapping with H3K9me3 peaks (Ward et al. 2018)
H3K9me3 peaks were identified and annotated as for H3K4me3 and H3K27me3 in this study, using sequencing reads obtained from Short Read Archive (SRA).

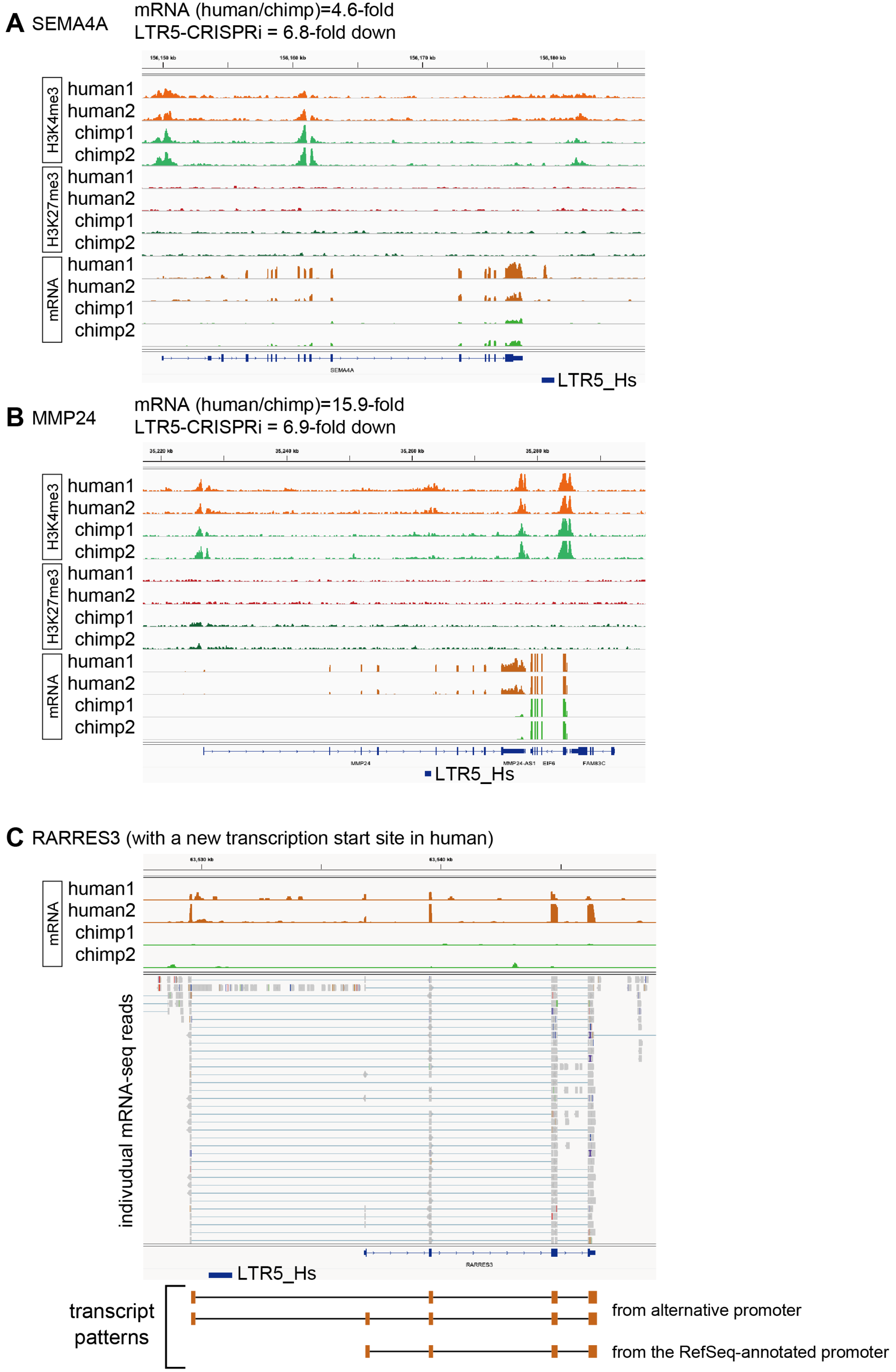

Figure S5. IGV snapshots of regions close to human-specific LTR5_Hs insertions

(A and B) Human-specific LTR5 insertions with H3K4me3 were associated with human-biased expression of SEMA4A and MMP24. (C) Individual mRNA sequencing reads around RARRES3 disclose a new transcription start site close to the human-specific LTR5_Hs copy. Splicing patterns are shown on the bottom. Most of detected transcripts were due to this new transcription start site. This region is also shown in Fig.5D.
